# Supplementary material for: Ultrafast 4D Scanning Transmission Electron Microscopy for Imaging of Localized Optical Fields
Source: ACS Photonics. 2025 Jul 21;12(8):4452–9. doi: 10.1021/acsphotonics.5c00864 (PMC12372171; doi:10.1021/acsphotonics.5c00864)
Supplement: Supplementary file 1 [file ph5c00864_si_001.pdf]

Supporting Information: Ultrafast 4D scanning transmission electron microscopy for imaging of localized optical fields

Petr Koutenský [a], Neli Laštovičková Streshkova [a], Kamila Moriová [a], Marius Constantin Chirita Mihaila [a], Alexandr Knápek [b], Daniel Burda [b], Martin Kozák [a]*

[a] Department of Chemical Physics and Optics, Faculty of Mathematics and Physics, Charles University, Ke Karlovu 3, Prague CZ-12116, Czech Republic.

[b] Institute of Scientific Instruments of the Czech Academy of Sciences, Královopolská 147, Brno, CZ-61200, Czech Republic.

* Email: petr.koutensky@matfyz.cuni.cz

* Email: m.kozak@matfyz.cuni.cz

13 pages, 5 figures, 0 tables

Experimental setup

The experimental setup is shown in Figure 1a. The electron-light interactions are studied in a scanning electron microscope Verios 5 UC (Thermo Fisher Scientific), which is modified for ultrafast operation. The electrons are photoemitted from the Schottky-type source using ultraviolet femtosecond laser pulses at the wavelength of 257 nm generated using the fourth harmonic generation of the output of Ytterbium-60 HE (AFS) femtosecond laser system with central wavelength of 1030 nm, pulse duration of 250 fs and repetition rate of 500 kHz. The fundamental output of the laser is used to excite the optical near-fields and to generate the optical standing wave inside the vacuum chamber of the microscope. All experiments are performed with electron kinetic energy of 20 keV. To allow sufficient transverse momentum resolution, we use the microscope setting that generates a beam with a low divergence angle of 1.3 mrad using the highest current settings and introducing an objective aperture with diameter of 64 μm. Each electron pulse contains approximately 0.038 electrons on average, as calculated from the laser repetition rate, exposure time, and number of detected electrons. The duration of the electron pulse in the sample plane was 520 fs (FWHM), see Figure 1e. The working distance is set to 11.5 mm. The spatial resolution of U4DSTEM of 21 nm is determined from the $35\% - 65\%$ contrast change in the bright-field STEM image of the nanotip, which is fitted by the error function.

The Gaussian width of the laser focus was $w_0 = (25 \pm 1)$ μm (FWHM) in the experiment with the nanotip and $w_0 = (8.8 \pm 0.1)$ μm in the experiment with the optical standing wave. The laser spot size is measured by scanning the nanotip through the laser focus and detecting scattered light. The time delay between the electron pulse and optical fields in the sample plane is controlled using an optical delay line (two independent delay lines are used in the experiment with the optical

standing wave). The intensity of excitation is controlled by a combination of a half-wave plate and a polarizer for both the fundamental and the photoemission beams. The electron detector (hybrid pixel detector Timepix3, Advascope) is placed at a distance of $L = 16.7$ cm downstream of the sample plane. The acquisition of the detector data is synchronized with the position of the electron beam in the sample plane, which is controlled externally using a PCIe-6323 card from National Instruments.

Preparation of nanotips

The preparation of sharp tungsten tips using the drop-off method on a polycrystalline tungsten wire with a diameter of 0.3 mm is based on the general technique of anodic dissolution [56]. The method comprises two etching phases: in the first phase, a narrowing of the tungsten wire, referred to as the meniscus, is formed. In the second phase, the tip is etched within a precisely defined region of this narrowing. Several variables influence the etching process, including the applied DC voltage, the type and concentration of the electrolyte (commonly a 8–10% NaOH solution) and the method's control parameters, such as the current limits set for each phase and the immersion depth of the wire below the electrolyte surface.

Since etching occurs preferentially where the liquid's surface tension exerts force on the wire, this method allows direct control over the meniscus region where the tip will be refined in the second phase. At the end of the second phase, the etching source is disconnected immediately after the bottom part of the meniscus drops off, based on the monitoring the second derivative of the electric current, to prevent tip blunting. This approach produces tips with diameters that typically range between 70 and 140 nm.

Data acquisition and processing

In the experiments, the pulsed electron beam is scanned across the sample and an image is taken on the detector placed downstream the sample. Figure 1d illustrates typical experimental data in the case of the interaction with optical near-field excited by light linearly polarized along the nanotip symmetry axis (perpendicular to the electron beam). The images shown in Figure 2a,c,f are measured with an acquisition time of 0.2 s per pixel, which gives the total measurement time of about 10 minutes for Figure 2a,c and 35 minutes for 2f (including readout time). Near-field distribution excited by light linearly polarized perpendicular to the tip axis (along the electron beam propagation direction) yielded suppressed scattering of the electrons (see Figure S1b,d) due to the lack of the field enhancement at the tip apex. A weak light-induced scattering is still present, likely because of a small residual ellipticity of the incident light polarization. In the classical point-particle approximation, the Lorentz force of the optical near-field deflects the electrons depending on the amplitude and phase of the oscillating field, which the electron experiences. The transverse momentum change of the electron $\Delta p_{\perp}$ depends on the phase of the optical field φ_0 and on the actual amplitude of the laser pulse envelope $g(\Delta t - t_0)$ during the interaction. Because the electrons are normally distributed within the electron pulse envelope, which is longer than the envelope of the laser pulse exciting the near-field and significantly longer than its cycle, the electrons experience a broad range of interaction strengths. As a result, the electrons scatter and form an elliptically smeared pattern on the detector, which we use to calculate the maximum transverse momentum change of the electrons as a function of the beam position in the sample plane. We note that the durations of both the electron pulse and the light pulse are much longer than the cycle of electric field oscillations. Therefore any effects related to the absolute phase (carrier-envelope phase) of the optical pulse are neither expected, nor observed.

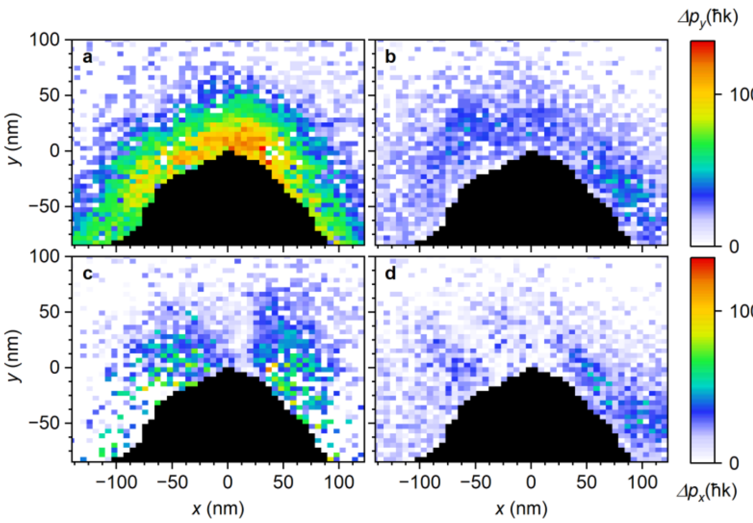

Figure S1. Transverse momentum change of the electrons Δp_y (**a, b**) and Δp_x (**c, d**) induced by the transverse component of Lorentz force of optical near-field generated on the surface of a tungsten nanotip by coherent optical excitation linearly polarized **a, c** along the nanotip axis (perpendicular to the electron beam) and **b, d** perpendicular to the nanotip axis (along the electron beam propagation direction).

The local direction and amplitude of the electron momentum change is extracted from the measured data by the following procedure. First, we filter out the electrons detected in the region of the primary beam and take into account only the electrons deflected out of this region. We note that we observe only a negligible shift of the primary beam on the detector with the U4DSTEM field of view of a few hundreds of nanometers. The azimuthal angle α of the electron deflection in the *x*-*y* plane is obtained by fitting the detected elliptical pattern with a 2D Gaussian function:

$$f(x_{\mathrm{det}}, y_{\mathrm{det}}, \alpha) = A\exp(-a x_{\mathrm{det}}^2 - 2b x_{\mathrm{det}} y_{\mathrm{det}} - c y_{\mathrm{det}}^2),$$

$$a = \frac{\cos^2(\alpha)}{2\sigma_1^2} + \frac{\sin^2(\alpha)}{2\sigma_2^2},$$

$$b = -\frac{\sin(\alpha)\cos(\alpha)}{2\sigma_1^2} + \frac{\sin(\alpha)\cos(\alpha)}{2\sigma_2^2},$$

$$c = \frac{\sin^2(\alpha)}{2\sigma_1^2} + \frac{\cos^2(\alpha)}{2\sigma_2^2}, \quad \text{(S1)}$$

where x_{det} and y_{det} are coordinates in the detector plane, σ_1 and σ_2 are the widths of the main and the secondary axis ($\sigma_1 > \sigma_2$), respectively, and α is the angle of rotation of the 2D Gaussion in the plane $x_{\mathrm{det}} - y_{\mathrm{det}}$ plane. The coordinate system is centered in the center of the electron beam on the detector. Figure S2a-c depicts 3 sets of raw data of elliptically smeared electron patterns detected on the detector. Following the above-described procedure, the data are fitted with 2D gaussian, FWHM of which is depicted by the red ellipse in the figures. From the fit we extract the value of α. Figure S2d, e show the comparison between the number of deflected counts from Figure S2b and the fit, both integrated over y_{det}, respectively x_{det}-axis. Other fitting functions like 2D Lorentzian or a description using covariance matrix may be used as well to determine the angle of the maximum momentum change α. Simulations (Supporting Information, section Numerical simulations) indicate that the shape of the deflected electron distribution does not follow an analytical function of the electric field. Nonetheless, the simulations reveal a correlation between the amplitude of the induced transverse momentum change of the electrons and the total number of scattered electrons.

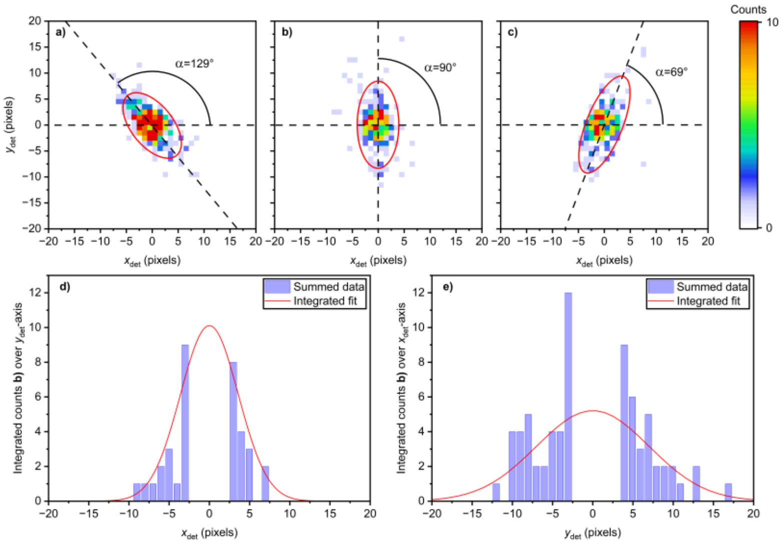

Figure S2. Examples of scattering patterns of electrons induced by deflection due to the interaction with the optical near-field on the detector. 2D Gaussian fits are used to determine the angle of scattering α **a, b, c** raw data detected by the detector fitted by 2D Gaussian, FWHM of which is depicted as the red ellipse. **d, e,** comparison between the number of deflected counts form **b** and the fit, both integrated over y_{det}, respectively x_{det}-axis.

Therefore, to determine the amplitude of the induced transverse momentum change, we first perform numerical simulations of the interaction by calculating the 3D distribution of optical near-field of a tungsten nanotip using a commercial finite-difference time-domain (FDTD) solver Lumerical FDTD. In the simulations we take into account the divergence and 3D envelopes of the electron pulse and the time envelope of the laser pulse used to excite the near-field. From the calculated distribution of the scattered electrons we determine the theoretical dependence of the number of electrons deflected out of the primary beam region on the detector on the maximum transverse momentum change $n(|\Delta\mathbf{p}_\perp|)$. The function $n(|\Delta\mathbf{p}_\perp|)$ is monotonous and its inverse function $|\Delta\mathbf{p}_\perp|(n)$ is used to determine the amplitude of the momentum change from the measured number of electrons scattered out of the primary beam region (details are described in the following

section). The background signal of images shown in Figure 2a, c, f at time delay $\Delta t = 13$ ps between the laser and electron pulses was negligible. Figure S3a, b. shows a different tungsten nanotip at $\Delta t = 0$ ps and $\Delta t = 13$ ps. Most of the elastic scattering at $\Delta t = 13$ ps occurs close to the edges of the nanotip.

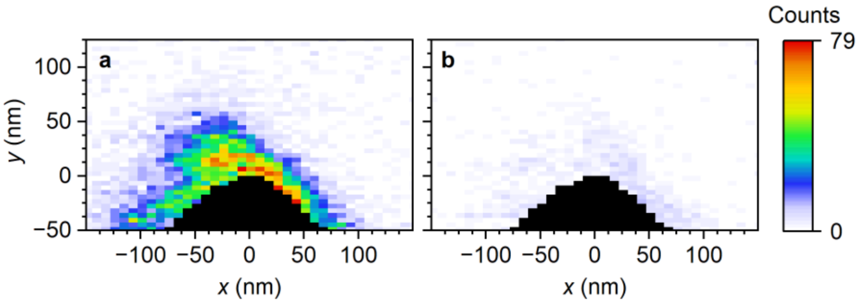

Figure S3. The total number of detected scattered electrons as a function of electron beam position close to the surface of a tungsten nanotip. **a,** at $\Delta t = 0$ ps. **b,** at $\Delta t = 13$ ps.

Due to the high amplitude of the electric field on the tip surface, multiphoton emission of electrons can be expected. Shortly after photoemission, a quasi-DC field is formed between the positively charged tip and the negatively charged electron cloud close to the tip surface. The effects of this quasi-DC field, whose dynamics is comparable or slower than the duration of the electron pulse [57] can be separately extracted from the U4DSTEM data by calculating shifts of the center-of-mass of the complete electron distribution on the detector. Figure S4 shows the background corrected shifts of the electron beam around the nanotip calculated from the center-of-mass motion of the electron beam at the detector from the same dataset as shown in Figure S3. Electron beam is pulled towards the nanotip, which suggests a presence of a transient quasistatic electric field. To extract the approximate charge of the cloud we assume a simple model of two static oppositely charged point particles and an electron passing in the center between them. When the calculated

electron deflection is compared with the measured values, the total charge in the electron cloud is estimated to be $Q \approx 40 \pm 10\ e$. The maximum observed beam shift of $\pm$ 0.5 pixels has negligible effect on the near-field processing, where we process the electrons scattered from the original beam spot by more than 1 pixel. The maximum deflections of the electrons caused by the oscillating optical near-field are more than order of magnitude larger than the maximum of the observed quasi-DC deflection and they can be separated from the center-of-mass motion of the electron beam due to their symmetry with respect to the electron beam axis.

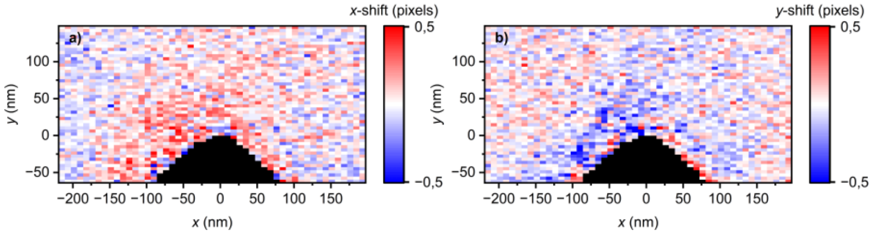

Figure S4. Center of mass processing of 4DSTEM data from Figure S3 to show the effects of quasi-DC field generated by a photo-emitted electron cloud on the electron beam **a,** center of mass shift along x-axis **b,** center of mass shift along y-axis. The shifts are aligned in the direction of the respective axis. The electron beam is attracted towards the tungsten nanotip indicating the presence of charge.

Numerical simulations

The evolution of electric and magnetic fields in the vicinity of a tungsten nanotip illuminated by a Gaussian pulsed beam with central frequency ω and linear polarization perpendicular to the tip surface at the apex (parallel to the tip symmetry axis) is calculated by numerically solving Maxwell's equations using FDTD. Because the relative spectral width of the laser pulses used in the experiments is only $\Delta\omega/\omega \approx 0.013$, we calculate the spatial distribution of the complex

amplitudes of the near-fields $\tilde{\mathbf{E}}(\mathbf{r},\omega), \tilde{\mathbf{B}}(\mathbf{r},\omega)$ generated at frequency ω using the Fourier transform of the time domain field. The simulation region has size of $5\ \mu\text{m} \times 5\ \mu\text{m} \times 16.235\ \mu\text{m}$ with perfect matching layer at all boundaries. As a source we use a Gaussian beam with the $1/e^2$ radius of the intensity of 2.5 μm. We note that the smaller beam size than the one used in the experiments is chosen due to the limitations of the 3D FDTD simulations. However, it does not influence the resulting near-field nor the elastic scattering of the electrons because the interaction is localized on much smaller spatial scales of only a few hundred nanometers. Adaptive mesh with the smallest step of 2 nm is used. The tip is modeled using the radial loft of the bright-field STEM image of the nanotip. The dimensions and shape of the tip apex were extracted from the bright-field 4DSTEM image. Figure S5 shows the renders of the tip. The dashed orange line in Figure S5b is an assumed plane of symmetry of the tungsten nanotip. The deflection corresponding to each electron trajectory is calculated in the classical approximation by using the transverse momentum change obtained from Eq. (2) with the Lorentz force given by the time-domain fields $\mathbf{E}(\mathbf{r},t) = \Re\{\tilde{\mathbf{E}}(\mathbf{r},\omega)g(t-\Delta t)\exp(i\omega t + i\varphi_0)\}$ and $\mathbf{B}(\mathbf{r},t) = \Re\{\tilde{\mathbf{B}}(\mathbf{r},\omega)g(t-\Delta t)\exp(i\omega t + i\varphi_0)\}$.

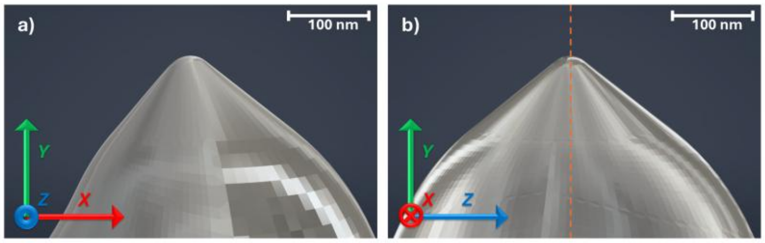

Figure S5. Renders of the model of the nanotip. **a**, view along *z*-axis, which matches the view in Figure 2b, d. **b** view along *x*-axis. The dashed orange line represents assumed plane of symmetry.

The electrons are propagated through the calculated electromagnetic fields $\mathbf{E}(\mathbf{r}, t)$ and $\mathbf{B}(\mathbf{r}, t)$ using the Monte-Carlo method. The initial electron distribution follows the properties of the electron beam in the experiments. We use normal distribution in time with FWHM duration of 520 fs, homogeneous distribution in the transverse coordinate in the sample plane with the diameter of 21 nm and randomly distributed polar angle in the region 0-1.3 mrad. We note that the relation between the maximum momentum change and the number of scattered electrons in each pixel in the sample plane $|\Delta \mathbf{p}_{\perp}|(n)$ is practically independent of the shape of the near-fields, providing that the ellipticity of the local Lorentz force is negligible for every *x-y* coordinate in the sample plane (electrons are scattered by the oscillating optical near-field approximately along one transverse direction). This assumption was verified by the simulations for our experimental conditions (linear polarization of the excitation, simple geometry of the nanotip). The shape of the electron scattering pattern on the detector and the associated functional dependence $|\Delta \mathbf{p}_{\perp}|(n)$ is determined by the combination of the electron beam parameters (width on the detector, divergence angle, pulse duration) and excitation laser pulse parameters (duration, frequency). This can be verified when we assume that the number of electrons scattered out of the primary beam region can be expressed as:

$$n = \int_{-\infty}^{\infty} b(\mathrm{t}) \int_{S_1} \int_{S_2} f(\mathbf{r}_{1\,\mathrm{det}}, \mathbf{r}_{2\,\mathrm{det}}, \mathrm{t})\, \mathrm{d}S_1\, \mathrm{d}S_2 \mathrm{d}t. \qquad \text{(S2)}$$

Here $b(t)$ is the electron pulse envelope, S_1 is the area at the detector covered by the incident unscattered beam, S_2 is the area on the detector in which we detect the scattered electrons, and $f(\mathbf{r}_{1\,\mathrm{det}}, \mathbf{r}_{2\,\mathrm{det}}, t)$ is a function describing the probability of electron scattering from coordinate $\mathbf{r}_{1\,\mathrm{det}}$ to $\mathbf{r}_{2\,\mathrm{det}}$ at the detector in time t, which can be approximated as:

$$f(\mathbf{r}_1, \mathbf{r}_2, t) = \frac{1}{\sqrt{1-\xi(t)^2}} \delta(y_{1\,\mathrm{det}} - y_{2\,\mathrm{det}}), \qquad \text{(S3)}$$

where $\xi(t) = (x_{1\,\mathrm{det}} - x_{2\,\mathrm{det}})\mathrm{p}_{\parallel}/\{|\Delta\mathbf{p}_{\perp}|Lg(t)\}$. The number of detected scattered electrons thus directly reflects the maximum momentum change $|\Delta\mathbf{p}_{\perp}|$, while it is not directly related to the distribution of the electric field $\mathbf{E}(\mathbf{r},t)$ but rather the integral of the field over the interaction time. The maximum transverse momentum change is an analogy of the coupling constant *g*, which is used to describe the near-field distribution in PINEM-type experiments.

The uncertainty of U4DSTEM in determination of the maximum transverse momentum change is limited by several factors. Besides the experiment geometry and spatial resolution of the detector it is limited by the statistical noise of the detected electron number. The standard statistical error of the number of detected electrons which are deflected out of the region illuminated by the undeflected beam *n* scales as $\sigma_n \approx 1/\sqrt{n}$. The standard error of maximum transverse momentum change can be expressed as $\sigma_{|\Delta\mathbf{p}_{\perp}|} = \left.\frac{\partial|\Delta\mathbf{p}_{\perp}|(N)}{\partial N}\right|_{N=n}\sigma_n$. For the region with maximal $|\Delta\mathbf{p}_{\perp}|$ in Figure 2a, c the standard error is approximately $\sigma_{|\Delta\mathbf{p}_{\mathrm{x}}|} \approx \sigma_{|\Delta\mathbf{p}_{\mathrm{y}}|} \approx 8\ \hbar k$. The signal/noise ratio and the statistical uncertainty in determination of maximum transverse momentum change can be improved by increasing the total amount of detected electrons. However, increasing the number of electrons per pulse is limited by the onset of Coulomb interaction leading to broadening the electron energy spectrum and pulse duration. The average electron current can be increased by increasing the repetition rate of the experiment.

Data processing - imaging of the optical standing wave

The shift of the center of mass of the deflected electron distribution, which is directly proportional to the induced ponderomotive force, is calculated for each position of the beam in the

sample plane. This processing, not applicable to process the optical near-field images, allows to achieve higher deflection sensitivity than is the angular size of individual detector pixels, 0.33 mrad. The maximum induced momentum change $\Delta\mathbf{p}_{\perp}$ is directly obtained from the highest value of the deflection angle of detected electrons (measured from the edge of the primary beam). The linear shift of the center of mass due to the large field of view was subtracted from the data. The intensity of the optical standing wave is calculated using Eq. (2) with the ponderomotive force given by Eq. (4).

We note that the concept of ponderomotive potential is not applicable in the case of the interaction of electrons with the optical near-fields of the nanotip. Assuming 20 keV electrons travelling in the distance of 100 nm from the tip apex, the for the effective interaction time between the electron and the field is approximately 1.2 fs, which is much less than one period of the oscillation of the excitation light of 3.4 fs. For this reason, the ponderomotive potential, which represents a cycle-averaged kinetic energy of a charged particle, does not represent a good model and does not influence the observed transverse electron scattering.
